# On Facing the SARS-CoV-2 (COVID-19) with Combination of Nanomaterials and Medicine: Possible Strategies and First Challenges

**DOI:** 10.3390/nano10050852

**Published:** 2020-04-28

**Authors:** Vishnu Sankar Sivasankarapillai, Akhilash M. Pillai, Abbas Rahdar, Anumol P. Sobha, Sabya Sachi Das, Athanasios C. Mitropoulos, Mahboobeh Heidari Mokarrar, George Z. Kyzas

**Affiliations:** 1 Department of Chemistry, NSS Hindu College, Changanacherry, Kerala 686102, India; vishnusankar005@gmail.com; 2 Department of Chemistry, University College, Thiruvananthapuram, Kerala 695034, India; akhilashm5@gmail.com; 3 Department of Physics, University of Zabol, Zabol 98615538, Iran; 4 Department of Biochemistry, University of Kerala, Thiruvananthapuram, Kerala 695581, India; anumolps1995@gmail.com; 5 Department of Pharmaceutical Sciences and Technology, Birla Institute of Technology, Mesra, Ranchi, Jharkhand 835215, India; sabya2049@gmail.com; 6 Department of Chemistry, International Hellenic University, 65404 Kavala, Greece; amitrop@chem.ihu.gr; 7 Department of English Language, Education office of Zahedan District 2, Zahedan 9817888741, Iran; m.heidarimokarrar@gmail.com

**Keywords:** COVID-19, SARS-CoV-2, coronavirus, medicinal chemistry, theranostic strategies, public health

## Abstract

Global health is facing the most dangerous situation regarding the novel severe acute respiratory syndrome called coronavirus 2 (SARS-CoV-2), which is widely known as the abbreviated COVID-19 pandemic. This is due to the highly infectious nature of the disease and its possibility to cause pneumonia induced death in approximately 6.89% of infected individuals (data until 27 April 2020). The pathogen causing COVID-19 is called severe acute respiratory syndrome coronavirus-2 (SARS-CoV-2), which is believed to be originated from the Wuhan Province in China. Unfortunately, an effective and approved vaccine for SARS-CoV-2 virus is still not available, making the situation more dangerous and currently available medical care futile. This unmet medical need thus requires significant and very urgent research attention to develop an effective vaccine to address the SARS-CoV-2 virus. In this review, the state-of-the-art drug design strategies against the virus are critically summarized including exploitations of novel drugs and potentials of repurposed drugs. The applications of nanochemistry and general nanotechnology was also discussed to give the status of nanodiagnostic systems for COVID-19.

## 1. Introduction

As from the beginning of 2020, the global health sector is passing through a perilous situation owing to the ongoing outburst of a novel severe acute respiratory syndrome called coronavirus 2 (SARS-CoV-2) [1]. This pandemic is abbreviated as COVID-19 (Coronavirus Disease 2019), as is occurred in Wuhan, Hubei province, in 31 December, 2019 and rapidly spread throughout China [2,3,4]. At the time of this writing, COVID-19 has spread most of the countries around the globe and it may have badly affected millions of people. Data updated as of 27 April, 2020, count 210 countries and territories around the world and one international conveyance (the “Diamond Princess” cruise ship harbored in Yokohama, Japan) with around 3,004,926 cases and 207,262 confirmed deaths (data until 24 April 2020) [5]. This virus appeared as a novel human pathogen that causes severe acute respiratory syndrome (SARS). According to the World Health Organization (WHO), there is incessant emergence of viral diseases, which causes serious issues to public health and medical communities [6].

In the past 18 years, several viral diseases have been reported, such as severe acute respiratory syndrome coronavirus (SARS-CoV) in 2002 to 2003, and H1N1 influenza in 2009. In 2012, Middle East respiratory syndrome coronavirus (MERS-CoV) was reported for the first time in Saudi Arabia [7,8]. In the beginning of the COVID-19 spread, it was not possible to identify the causative agent, and the first cases were reported as “pneumonia of unknown etiology”. Later, The Chinese Center for Disease Control and Prevention (CDC) and local CDCs conducted rigorous outbreak research programs. The etiology of this disease is now identified to a new virus belonging to the coronavirus (CoV) family. Compared with the SARS-CoV in 2002, this new virus has a much stronger communicable capacity and has hastily spread globally. On 30 January 2020, the outbreak of SARS-CoV-2 was declared by the WHO as a Public Health Emergency of International Concern (PHEIC) because it had spread to 18 countries and because four countries reported human-to-human transmission [9].

As we know, genetic information is usually carried by DNA, however, for many viruses including HIV and the influenza virus, RNA is used as their basic genetic material. The corona viruses also belongs to the RNA virus family and it consists of a single, 30,000-base-long RNA [10]. A large family of single-stranded RNA viruses (+ssRNA) can be isolated from different animal species [11]. These viruses can cross species barriers and in humans it can cause illness ranging from the common cold to more severe diseases such as SARS and MERS. SARS viruses have possibly originated from bats and then transmitting into other mammalian hosts. For example, the Himalayan palm civet acts as intermediate for SARS-CoV, and the dromedary camel for MERS-CoV before transition to humans. The dynamics of SARS-Cov-2 are presently unknown, but there is conjecture that it also has an animal origin [12,13,14,15].

At present there are no vaccines, monoclonal antibodies (mAbs), or drugs available for COVID-19. However, a sturdy research effort is presently under way to develop a vaccine against COVID-19 and some may be available in a short time [6]. Currently, most of the countries have taken strategies such as social distancing in order to reduce community spread. Such approaches could include isolating ill persons (including voluntary isolation at home), school closures, and telecommuting where possible [16].

Kampf et al. conducted a systematic review on the analysis of literature regarding different human corona viruses such as Severe Acute Respiratory Syndrome (SARS) coronavirus, Middle East Respiratory Syndrome (MERS) coronavirus, or endemic human coronaviruses (HCoV). The authors concluded that these viruses can persist on inanimate surfaces such as metal, glass, or plastic for up to 9 days. Further they can be effectively eliminated by using surface disinfection procedures with 62% to 71% ethanol, 0.5% hydrogen peroxide, or 0.1% sodium hypochlorite within 1 min [17].

This article summarizes the state-of-the-art drug design strategies against the COVID-19 virus including exploitations of novel drugs and potentials of repurposed drugs. The applications of nanochemistry and generally nanotechnology were also discussed to give the status of nanodiagnostic systems for COVID-19.

## 2. New SARS-CoV-2 (COVID-19)

### 2.1. Society Impact, Diagnosis, Treatment Approaches

The World Health Organization (WHO) announced “COVID-19” as the official name for the 2019 novel CoV, a CoV variety that infected and distressed the lower respiratory tract of patients with pneumonia in Wuhan, China. Additionally, it has been provided with reference names such as SARS-CoV-2. In the fall 2019, a group of patients were reported with pneumonia of indefinite cause with association to a native Huanan South China Seafood Market in Wuhan, Hubei Province, China, after which it has spread globally [18,19,20]. As compared to SARS-CoV, the COVID-19 has showed enhanced levels of pandemic and transmission risk, as the effective reproduction number (R) of COVID-19 or SARS-CoV-2 (2.9) was found to be more than the earlier reported effective reproductive number (R) of SARS-CoV (1.77) during the early stage [21]. Results from various laboratories have also revealed that COVID-19 exhibited similar pathogenesis and behavioral conditions as that of beta-CoV genera recognized in bats, which was located in a cluster of SARS or SARS-like CoV [22].

To date, complete and comprehensive clinical expression for COVID-19 or SARS-COV-2 is not distinct and clear, as the reported and observed symptoms and indications in infected patients ranges from mild to severe, even resulting in death for some [19]. From the study cases reports of till date, fever, cough, pneumonia, myalgia or fatigue, and complex dyspnea were found to be some of the most common symptoms, however, headache, diarrhea, runny nose, hemoptysis and phlegm-producing cough were reported as less common symptoms [9,19]. It was reported that the patients with mild symptoms improved their health conditions after one week while the patients with severe infections due to virus experienced progressions in respiratory tract most likely due to the alveolar injury and ultimately lead to death [23]. Medical experts and virologists have suggested the patients with suspected infections to follow the diagnosis procedures: execution of RT-PCR to recognize the positive nucleic acid of COVID-19 in sputum, swabs from throat and secretions of the lower respiratory tract samples.

As far as the reports have showed, no specific and exact antiviral treatment has been established yet for the effective treatment or management of COVID-19. Concerning the COVID-19 infected patients, it has been suggested to follow suitable symptomatic management and supportive cautions [9,19]. For the evaluation of the efficiency or safety of the targeted medication for the prognosis of COVID-19, six clinical trials have been registered in both the Chinese Clinical Trial Registry and the International Clinical Trials Registry [20,24]. Moreover, as this severe pandemic spreads worldwide, no vaccine has been developed yet for the prevention or management of COVID-19. So far, the preeminent approach for prevention from this pandemic virus is to circumvent ourselves from being exposed to this viral infections [25].

The treatment with established antiviral drugs and general corticosteroids used for general clinical practice including neuraminidase inhibitors (oseltamivir, zanamivir, peramivir, etc.), acyclovir, ganciclovir and ribavirin, as well as methylprednisolone for influenza virus, have found to be inacceptable for the prevention and treatment of COVID-19 and also are not recommended [26]. Moreover, remdesivir (GS-5734), a 1′-cyano-substituted adenosine nucleotide analog prodrug, have exhibited potent antiviral activity against numerous RNA viruses. In one of the reported case in US, being the first case reported till date, remdesivir usage effectively treated COVID-19 infected patient [27].

On the other hand, recent publications have disclosed that chloroquine (CQ) can potentially inhibit the in vitro replication of some CoVs. So, the experts hypothetically suggested that CQ can progress the clinical consequences of the COVID-19 infected patients. However, the molecular mechanisms by which CQ could accomplish such outcomes remains to be further examined and explored. Since, COVID-19 was established a few days ago, to utilize the similar cell surface receptor ACE2 against SARS-CoV-1 [28,29], it might be hypothesized that CQ could potentially inhibit the glycosylation of the ACE2 receptor and thus could prevent the COVID-19 or SARS-CoV-2 binding to the targeted cells. Certainly, if COVID-19, as other CoVs (beta-CoVs), targets the sialic acids on some cell subtypes, then CQ could hinder the interactions and thus could be able to treat the viral infections [30,31]. Today, primary studies have indicated that CQ interfere with COVID-19 efforts to acidify the lysosomes and apparently hinders the cathepsins, which requires a low pH for optimum cleavage of COVID-19 spike protein, a prime necessity to the establishment of the auto-phagosome [32,33]. It could be suggested that a combination of CQ and remdesivir could be a potent therapy for the management of pandemic COVID-19 in vitro.

ARDS (acute respiratory distress syndromes) are distressing conditions of prodigious pulmonary inflammations and hypoxemic circumstances, leading to severe illness and mortalities. The key therapeutic approaches have been concentrated over various strategies that could efficiently inhibit extreme inflammations or managing the subsequent physiological imbalance, triggering respiratory lapse [34]. There are various therapeutic interventions which are currently used in patients suffering from ARDS, including anti-inflammatory agents (corticosteroids, pharmaconutrients, antioxidants, anti-proteases, ketoconazole), ventilator agents (neuromuscular blockers, β2 agonists, surfactants), diuretics, anti-coagulants, vasodilators and others. Apart from these approaches, various emerging therapies have also been reported, which includes anti-inflammatory agents (statins, insulin, macrolides, MMPs, aspirin, vitamin D, anti-interleukin 8), cellular therapies (stem cells, growth factors, colony stimulating factors) angiotensin-converting enzyme (ACE) inhibitors and forth more [35]. Moreover, in 2002, the importance of the ACE-2 enzyme was found seemingly widespread in the case of severe acute respiratory syndrome (SARS). Interestingly, for a novel coronavirus infection that caused impaired functions, unobstructed functioning of renin-angiotensin system (RAS) and thus caused acute lung injuries, the ACE-2 enzyme was recognized as a specific receptor [36]. Furthermore, such strategies could possibly decrease the incidence of mechanical ventilation injuries, so preclinical or clinical trials focusing hindrance and initial interventions should be accessed to mitigate ARDS.

### 2.2. Pathophysiology and Related Facts

Coronavirus possesses a positive-sense single-stranded-RNA genome. The angiotensin responsible in converting enzyme 2 (ACE2) has been recognized as the host cellular receptor for SARS-CoV2 envelope spike glycoprotein [37]. The type I membrane protein, ACE2, particularly expressed on cells in the heart, blood vessels, kidney, gastrointestinal tract, and, most importantly, lung AT2 alveolar epithelial cells, are easily prone to viral infections [38]. SARS-CoV-2 infection leads to the down regulation of ACE2 expression, thus resulting in excessive production of angiotensin II by the related enzyme ACE. Pulmonary vascular permeability is increased by the stimulation of type 1a angiotensin II receptor (AGTR1A) and thus the decreased expression of ACE2 leads to an increased lung damage [39]. It has been postulated that, this type of mechanistic pathway causes an increased risk of infection as well as the harshness of COVID-19 [40]. When the viral RNA genome is discharged into the cytoplasm, and the RNA is uncoated to permit translation of transcription of the sub-genomic RNAs as well as for the replication of viral genome [41]. Progression to ARDS causes Cytokines Release Syndrome (CRS), that is, the upregulation of pro-inflammatory cytokines and chemokines, and this pattern, is very similar to that of secondary haemophagocytic lymphohistiocytosis (sHLH). Approximately around 50% patients’ sHLH causes unrelenting fever, cytopenias and hyperferritinaemia, and pulmonary involvement [42,43]. In the majority of severe COVID-19 infections, a sHLH-like cytokine profile has been reported. This cytokine profile is characterized by increased levels of a number of cytokines (interleukin-1β [IL-1β], IL-2, IL-6, IL-7, IL-8, tumor necrosis factor-α [TNF]) and chemokines (CXC-chemokine ligand 10 [CXCL10] and CC-chemokine ligand 2 [CCL2]) [44,45]. The administration of this cytokine tornado is one of the major challenges concerning COVID-19 infection.

## 3. Drug Development for SARS-CoV-2

Until now (unfortunately) there is no any effective recipe for drug synthesis or use for SARS-CoV-2 infection; the latter results in fatal inflammatory responses and acute lung injury. Effective and suitable drug candidates have to be designed to resolve this due to this urgent and unmet medical need. SARS-CoV-2 Mpro protease constitutes one of the most attractive antiviral drug targets, especially in the design and development of SARS drugs.

Rut et al. provided a structural framework for the design of inhibitors as antiviral agents or diagnostic tests by preparing a combinatorial library of fluorogenic substrates with glutamine in the P1 position [46]. The authors detected substrate specific preferences of the SARS-CoV and SARS-CoV-2 proteases, using natural and a large panel of unnatural amino acids.

Ton et al. developed a novel deep learning platform called “deep docking” (abbreviated as DD) and conducted docking studies on 1.3 billion compounds from ZINC15 library and identified the top 1000 potential ligands for SARS-CoV-2 Mpro protein [47]. These structures are made available to the research community for doing subsequent experiments involving cell culture and animal model experiments. This study is very relevant since it is very fast in obtaining docking score from in silico experiments and enables structure-based virtual screening of billions of purchasable compounds in a short time. DD relies on a deep neural network trained with docking scores of small random samples of molecules extracted from a large database to predict the scores of remaining molecules and, therefore, discard low scoring molecules without investing time and resources to dock them.

On the other hand, controversial inference which indicates the lack of suitability of SARS-CoV-2 Mpro as the protein target was also reported. Molecular dynamics investigations on Mpro with a highly similar SARS protein was also reported [48]. This study stated that the active sites in both proteins showed major differences in both shape and size indicating that repurposing small molecule inhibitor SARS drugs for COVID-19 may be a futile exercise. The neutralizing antibody, which binds to viral capsid to inhibit cellular entry of virus and uncoating of the genome, is the specific defense against viral pathogens.

Park et al. attempted an investigation to identify neutralizing antibodies that can bind to SARS-CoV-2 Spike (S) protein and interfere with the interaction between viral S protein and a host receptor by bioinformatics tools [49]. The sequence analysis of S protein showed two major differences in the RBD region of the SARS-CoV-2 S protein compared to SARS-CoV and SARS-CoV related bat viruses (btSARS-CoV). The insertion regions were close to interacting residues with the human ACE2 receptor. The authors successfully demonstrated that the CR3022 neutralizing antibody in human beings may have higher binding affinity with SARS-CoV-2 S protein than SARS-CoV S protein. Further, F26G19 and D12 mouse antibodies could bind to SARS-CoV S protein with high affinity.

Qamar et al. conducted an effective multi-epitope vaccine (MEV) against SARS-CoV-2 by taking seven antigenic proteins as target and epitopes (B cell, IFN-γ and T-cell) [50]. Docking studies demonstrated a stable and strong binding affinity of MEV with TLR3 and TLR8, while codon optimization and in silico cloning ensured increased expression in the *Escherichia coli* K-12 system. Future experimental validations in this direction can give valuable results in vaccine development.

Ko et al. reported 54 molecular hits with a therapeutic index (TI) greater than 6 by screening 5406 molecules including US Food and Drug Administration (FDA) approved drugs and bioactive compounds [51]. This study demonstrated that out of 12 FDA approved drugs, 8 and 4 therapeutics act on the early and late stages of the viral life cycle, respectively. Among the early acting drugs, 3 therapeutics with a TI > 100 were cardiotonic agents.

Zhang et al. suggested the potential of Chinese medicine to develop a drug against COVID-19 by in silico screening and network pharmacology of Chinese herbal medicines [52]. Zhu et al. introduced a ligand-based approach, named “D3Similarity”, which is based on the molecular similarity evaluation between the submitted compound structures and those in an active compound database [53]. This study revealed reliability and efficiency of D3Similarity based on the two-dimensional and three-dimensional similarity evaluation of molecular structures, virtual screening, and target prediction could be performed according to similarity ranking results.

**Figure 1:**
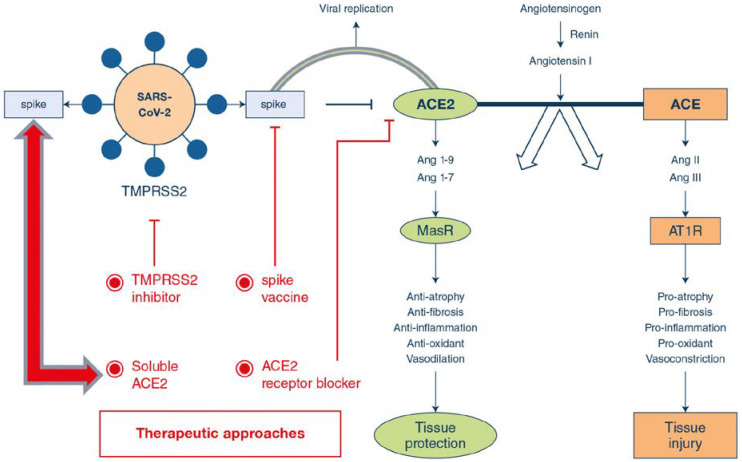
Pathophysiology and role of ACE2 based therapeutic mechanism of SARS-CoV-2 infection. Reprinted with permission taken by Springer [54].

Some other receptors were also reported as the therapeutic target for designing drugs against COVID-19. Zhang et al. reported angiotensin converting enzyme 2 (ACE2) receptor as a potential target [54]. It is based on the fact that cell lines that facilitate viral replication in the presence of ACE2 may be most efficient in large-scale vaccine production. The authors demonstrate four major therapeutic approaches regarding ACE2 receptor such as: (i) spike protein-based vaccine; (ii) inhibition of trans-membrane protease serine 2 (TMPRSS2) activity; (iii) blocking ACE2 receptor; and (iv) delivering excessive soluble form of ACE2. These approaches are schematically represented in Figure 1 given below.

## 4. Old Drug Recipes for the New Target

The concept of drug repurposing deserves significant attention in managing COVID-19 treatment owing to its extremely high infectious rate and urgency for the unmet medical need. Repurposed drugs are very beneficial since they can bypass the preclinical trials associated with drug research and can give quick benefit to patients [55]. Guo have summarized the importance of repurposed drugs in COVID-19 therapeutics and other viral diseases related to it [56].

Wang et al. evaluated FDA approved drugs including ribavirin, penciclovir, nitazoxanide, nafamostat, chloroquine (CQ) and two well-known broad-spectrum antiviral drugs remdesivir (RDV, GS-5734) and favipiravir (T-705) against a clinical isolate of 2019-nCoV in a cell culture infection model [33]. The authors found that two compounds CQ (EC_50_ value = 1.13 μmol/L; CC_50_ > 100 μmol/L, SI > 88.50) and RDV (EC_50_ = 0.77 μmol/L; CC_50_ > 100 μmol/L; SI > 129.87) potently blocked virus infection at low-micromolar concentration and showed high selectivity index. RDV is already proved to be active against several viruses and currently under clinical trials to evaluate its efficacy against Ebola virus infections. This study gives additional insights on the use of this adenosine analogue prodrug against COVID-19 infections. On the other hand, CQ is already well known for its action against malarial parasite infections and anti-inflammatory properties [57,58].

**Figure 2:**
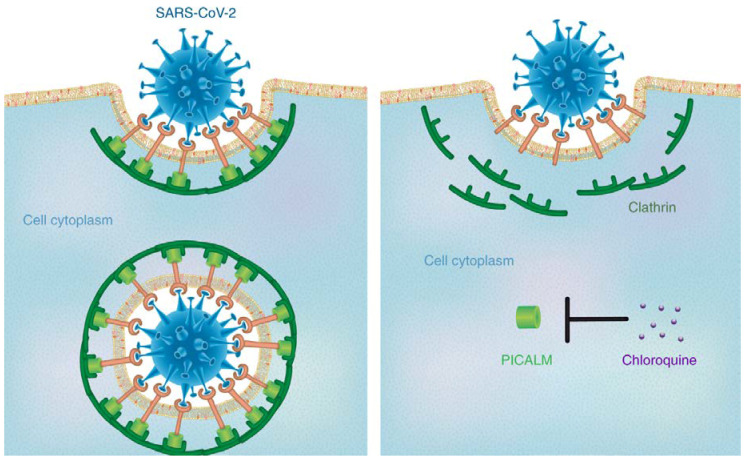
Mechanism of action of chloroquine against SARS-CoV-2. Reprinted with permission taken by Springer Nature [62].

Further CQ has been approved for the clinical treatment of autoimmune diseases such as lupus erythematosus and rheumatoid arthriti [59]. Recently, CQ have been revealed to suppress the infection of a diverse group of viruses including SARS-CoV, MERS-CoV, EBOV, influenza A virus, Chikungunya virus, human immunodeficiency virus, dengue virus, West Nile virus, Crimean Congo hemorrhagic fever virus, and hepatitis A virus [60]. Savarino et al. demonstrated that CQ can efficiently enter the cells and accumulate in acidic compartments such as lysosomes, endosomes and trans-Golgi network vesicles, consequently raising their pH value, while many viruses need the acidic endocytic organelles at some stages of their replication, such as viral uncoating and cellular entry via membrane fusion [61]. The detailed mechanism of action of CQ thus requires significant attention. Tony et al. provides the most possible mechanism of action of CQ against SARS-CoV-2, which is the suppression of phosphatidylinositol binding clathrin assembly protein (PICALM), which prevents endocytosis-mediated cellular uptake of SARS-CoV-2 [62]. This can be represented, as shown in Figure 2.

Gao et al. reported that Chloroquine phosphate was very effective and safe for COVID-19 associated pneumonia in multicenter clinical trials conducted in China [63]. The hydroxyl derivative of CQ, hydroxychoroquine (HCQ) was also very recently reported for its action against SARS-CoV-2. Zhou et al. suggested the efficacy of HCQ in attenuating the severe progression of COVID-19, inhibiting the cytokine storm by suppressing T cell activation and with an advantage of safety clinical profile especially suitable for pregnant women [64].

Gautret et al. successfully demonstrated the synergistic effect of hydroxychoroquine and azithromycin in 20 COVID-19 cases and observed efficient viral elimination [65]. Similarly, Xu et al. also reported the efficacy of broad spectrum antiviral agent Niclosamide against COVID-19 [66]. This work is also a significant example for drug repurposing extending the scope to clinical trials. Fan et al. used pangolin coronavirus GX_P2V as a workable model for evaluating the efficacy of repurposed drugs for 2019-nCoV treatment [67]. This study revealed that cepharanthine (CEP), selamectin, and mefloquine hydrochloride exhibited complete inhibition of cytopathic effects in cell culture at 10 μmol/L with CEP having most potent inhibition of GX_P2V infection, with a concentration for 50% of maximal effect [EC50] of 0.98 μmol/L. Hui et al. suggested the use of remdesivir, lopinavir/ritonavir, lopinavir/ritonavir combined with interferon-β, convalescent plasma, and monoclonal antibodies 2019-nCoV pneumonia patients [68].

Controversial results are indicated by Cao et al; lopinavir-ritonavir treatment did not resulted in any benefit beyond standard care [69].Wang et al. reported that there was significant improvement in patients who underwent antiviral treatment including lopinavir/ritonavir regarding their pneumonia associated symptoms [2]. This treatment also significantly reduced β-coronavirus viral loads. Russel et al. reported that corticosteroid treatment for 2019-nCoV lung injury is not effective [70]. All these observations suggest a thorough investigation for understanding the effects of conventional antiviral drugs against COVID-19.

Favalli et al. provides an indication for using antirheumatoid arthritis drugs for COVID-19 with their pros and cons by correlating with the pathophysiology of COVID infection [71]. The authors suggested following type of anti-rheumatoid arthritis drugs for possible effect towards COVID virus as shown in Table 1.

Liu et al. observed that an anticoagulant agent dipyridamole (DIP) suppressed HCoV-19 replication at an EC50 of 100 nM as evident from in vitro studies [72]. The authors selected DIP by screening an FDA approved drug library and concluded that HCoV-19 infected patients could potentially benefit from DIP adjunctive therapy by reducing viral replication, suppressing hypercoagulability, and enhancing immune recovery.

Sang et al. verified the assumption of using HIV-1 protease inhibitors as anti-SARS drugs by targeting SARS-Co-V 3CLpro [73]. The authors employed six approved anti-HIV-1 drugs to investigate their binding interactions between 3CLpro. Molecular docking and MM-PBSA binding free energy calculations demonstrated that Darunavir has the best binding affinity with SARS-Co-V-2 and SARS-Co-V 3CLpro among all inhibitors, indicating the potential to become an anti-COVID-19 drug.

Chen et al. did virtual screening using 3CL (pro) molecular model and observed that antivirals ledipasvir or velpatasvir are particularly attractive as therapeutics acting through dual inhibitory actions on two viral enzymes with minimal side effects [74]. Phytochemicals with antiviral effects should also get significant attention. Shaghaghi reported the effectiveness of terpenoids for the use as low risk drugs by doing molecular docking studies in novel COVID-19 protease [75]. Elfiky suggested the effectiveness of Sofosbuvir, IDX-184, Ribavirin, and Remidisvir as potent repurposed drugs against the HCoV disease, which is a good example of drug repurposing [76]. Baron et al. reported Teicoplanin, an antibiotic used to treat staphylococci infection as an alternative drug against SARS-Cov-2 affected patients [77]. This observation has to be confirmed through animal studies since it was already proved to be active against previous corona viruses.

## 5. Perspectives in Nanoscience against Respiratory Viruses

Advancements in nanoscience regarding viruses causing respiratory diseases can be found. Although there is not any literature available regarding drastic and successful therapeutic strategies of nanotechnology against COVID-19, the following data can guide the upcoming research works for developing both a successful and effective nanomedicine against SARS-CoV-2 and “give an obstacle” of related pulmonary disorders associated with these viral infections.

The advantages of using nanomedicine for the management of respiratory viruses were reviewed in literature [78]. The gateway of respiratory entry for most of the viruses including influenza and respiratory syncytial virus is the respiratory mucosa. Their journey to the lower respiratory tract after infecting the upper respiratory tract is the cause of respiratory diseases. Conventional and subunit vaccines possess limitations such as reversion to pathogenic virulence leading to weak immune response and partial or limited immunogenicity, respectively. These limitations can be resolved by the implementation of nanoparticles (NPs) based therapeutic approaches due to their features such as size and shape control and surface functionalization which ultimately leads to strong immunogenicity and enhanced antigen presentation. Recent literature suggests the toxicity concerns of NPs as quantum dots which need to be carefully addressed while designing theranostic NPs for respiratory diseases [79].

Novel NPs were reported, which can improve the performance in the treatment of respiratory diseases through different mechanism of action which are (i) the development of polymers with faster mucus penetration and do not remain stuck, overcoming this barrier, (ii) the creation of biodegradable NPs with the stability to overcome the cell membrane and act in the lung with minimal levels of toxicity, causing no lesions during treatment, (iii) modification of the chemical structure of NPs by adding surface capping agents such as polyethylene glycol (PEG).

### 5.1. Nanoscience to Face Various Viruses

Influenza virus: Genomic mutations and antigenic shifts between various influenza species yields a high degree of variance that leads to the inception of novel influenza viral strains as well as unnecessary drug resistance [80]. A polymeric nanosystem of STP702 (FluquitTM) derived from Sirnaomics is presently under preclinical investigations. The systems encapsulate siRNA that could target the conserved areas of influenza to display significant antiviral reaction against H1N1 (swine flu), H5N1 (avian flu), and the newly found H7N9 [81]. Heat stimulant hydrogels called ‘Nanotraps’ are successful in trapping living viral cell, RNA, and proteins [82]. This novel technology could be further employed for treating infectious disorders such as influenza virus. Hendricks et al. successfully employed liposomes for transporting glycan sialylneolacto-N-tetraose c (LSTc)- sialoside, a synthetically derived receptor to bind and capture the influenza A virus, in a dose dependent fashion [83]. Hemagglutinin (HA) as well as neuraminidase (NA), are influenza glycoproteins, which function in viral attachment (to sialic-acid containing receptors on the cell surface) and release, respectively [84] Oseltamivir, a NA inhibitor, impedes the cell-cell spread of influenza virus [85]. Li et al. modified silver NPs (AgNPs) with oseltamivir to proficiently reduce H1N1 infection by constraining both HA and NA, in vitro. Nanocarriers also demonstrated antiviral actions by DNA fragmentation prevention, condensation of chromatin and caspase-3 function. Toxicity studies of oseltamivir-modified silver nanoconstructs were identified by TEM, electron microscopy, cell viability assays, and cytopathic effect and were seen more than oseltamivir control drugs in MDCK cell lines [86]. In a different study, polylysine-linker was used to fabricate DNA functionalized titanium dioxide (TiO_2_) nanocarriers that targeted the 3′non-coding area of influenza a virus. These NPs entered cells without the aid of transfection agents and strongly inhibited influenza A in vitro [87].

Human papillomavirus: These viruses target the epithelial cells and yield a lot of symptoms, which include common warts, cervical carcinoma, and cancer. More than 100 types of HPV have been identified and classified to be highly risky STP909 (CervisilR), a nano drug candidate, is loaded with siRNA to combat HPV16 and HPV18, which account for 70% of cervical cancer. In vitro studies show that from the E7 genes of HPV16 and HPV18, mRNA duplexes were formed, while tests in rabbits demonstrated that the NPs result in a knock-down of E7 gene. Many other gene silencing experiments that target E7 gene in mice models, mammalian cells, as well as vaccine studies, have been investigated in vaccine formulations [88,89].

Respiratory syncytial virus (RSV): A siRNA loaded lipid based nanocarrier, ALN-RSV01, combats the nucleocapsid “N” gene which is a key viral protein of RSV. It was the first RNAi-based treatment authorized for clinical studies and has now reached phase II, which indicate very safe and successful antiviral consequences [90,91].

Human parainfluenza 3 (HPIV-3): The latest research revealed suppression of HPIV-3 replication, possibly due to a blocking function of the cell-virus leveraging AgNPs. The findings of this analysis indicate that the inhibitory behavior depends on both the NPs size and zeta potential [92].

Although, till date, no specific targeted delivery systems have been developed or explored for the management SARS-CoV, SARS-CoV-2 (COVID-19) and other CoV strains, it is expected that nanotechnology-based treatment approaches including monoclonal antibodies or vaccines, can find a way for efficient and rapid diagnosis.

### 5.2. Specific Examples of Nanoparticles against Viruses

**Figure 3:**
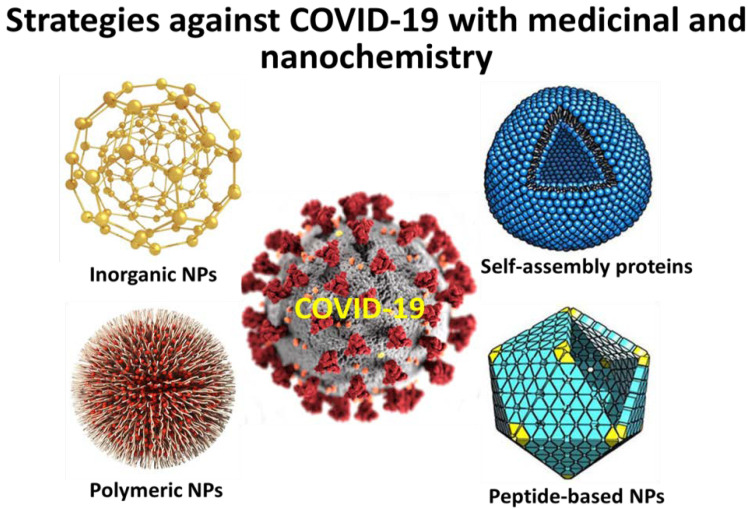
Applications of nanoscience to face COVID-19.

Disease caused by respiratory syncytial virus (RSV) was reported to be eliminated in mice by using virus-like NPs carrying RSV fusion proteins (F VLP). The advantage of using FVLP is that they act against RSV by inducing natural killer cells, activated IFN-γ(+), and IFN-γ(+) tumor necrosis factor (TNF)-α(+) CD8(+) T-cells in the lung and bronchiolar airways during the infection stage but not forming harmful lung plasmacytoid dendritic cells (DCs) and effector T-cells [93]. RSV was also successfully inhibited by 56% in BALB/c mice by gold nanorods through upregulated antiviral genes due to GNR mediated TLR, NOD-like receptor and RIG-I-like receptor signaling pathways. Figure 3 illustrate the major types of nanoparticles which are reported to act against viruses and can be promising tools to act against COVID-19 infections. 

Major NPs reported for acting against respiratory viruses are three categories given below [78].

Polymeric NPs: They possess exciting properties such as tunable properties, feasible synthetic protocols, and good biocompatibility, which makes them interesting candidates for biomedical applications. Poly (lactic-co-glycolic acid) (PLGA) is one of the well-known member of this category which is approved by FDA for application in human body. This is due to the excellent biocompatibility and biodegradability in the human body [94]. Other examples include chitosan, and N-(2-hydroxypropyl) methacrylamide/N-isopropylacrylamide (HPMA/NIPAM) which showed promising results as intranasal vaccines against respiratory viruses

Self-assembling proteins NPs: These are prepared through the oligomerization of monomeric proteins, which were found suitable for biomedical applications [95].

Inorganic NPs: Extensive literature is available regarding the biological effects of inorganic NPs such as metal oxide NPs, which showed good results in antibacterial and antifungal studies [96]. Features such as easy synthesis, biocompatibility, and optical properties make them suitable for biological applications. However, inorganic NPs that show good action against respiratory viruses are scarce and need to be investigated thoroughly with urgent attention.

Peptide-based NPs: Earlier studies have showed applications of peptide inhibitors (short-sequenced) and mutations of amino acids could potentially act against the infections associated with SARS-CoV [97]. As reported, a peptide-based vaccine which could express HRC, trimeric coil conformation stage, could act as an ideal therapeutic approach for the intervention of SARS-CoV associated infections, and this approach is established through peptide-based NPs [98]. In another study, a vaccine moiety, P6HRC1, was achieved by binding a peptide ligand with a B-cell epitope from the SARS-B HRC1 spike protein and self-accumulated through dialysis in presence of refolding buffer. Results showed that the researchers were able to generate the requisite conformation-specific antibodies which potentially neutralized the SARS-CoV infections via NPs-based systems [99]. In recent days, researchers are concurrently working on the establishment of potential peptide-based approaches on the basis of preliminary molecular dynamics simulation studies. In a recent study, a peptide inhibitor isolated from ACE2 provided significant traces for the blocking of SARS-CoV-2. Furthermore, it was reported that the binding efficacy can be increased through multiple binding of nanocarrier-linked peptides [100].

The reported NPs against different respiratory viruses through nasal administration are summarized in Table 2.

There exists a huge urgency for developing a successful nanovaccine in order to reduce mortality and hospitality currently faced by most of the countries. “LIF nano”, which is a special class of mesenchymal stem cells, are also reported to improve patient’s biological resistance to COVID-19 using stem cells [122]. These types of approaches are very optimistic since they can reduce the massive health deterioration occurring in pneumonia-affected patients as a part of COVID-19 infection. All above can be schematically represented in Figure 3.

## 6. Nanosensors

NPs-based biosensors are vital tools that contribute to the detection of individuals affected with pathogens. Theragnostic aspects of nanosystems as quantum dots were reviewed in recent literature [123]. Sensory systems were successfully reported for other microorganisms in literature. Unfortunately, such rapid and sensitive diagnostic systems for COVID-19 is limited and urgently demonstrated due to its longer incubation period and extremely higher infectivity. Conventional detection methods rely on nucleic acid detection and have various demerits which are (i) low sensitivity and laborious experimental procedures, (ii) large time duration between sample collection and results interpretation, (iii) high false negative rates, and (iv) lack of specificity which results in misdiagnosis in patients having other viral infections.

Zhu et al. reported a NPs-based biosensor system for COVID-19 which works via a “one step and single tube” reaction pathway [124]. This sensor employs a single step reverse transcription loop-mediated isothermal amplification (RT-LAMP) coupled with NPs-based biosensor (NBS) assay (RT-LAMP-NBS). Additionally, equipment for providing isothermal condition (63 °C) is needed for 40 min and the time duration for result interpretation from sample collection is only 1 h, approximately. This SARS-CoV-2 RT-LAMP-NBS biosensor was successfully demonstrated for COVID-19 confirmed patients with a promising sensitivity of 12 copies (each of detection target) per reaction with no cross-reactivity was generated from non-SARS-CoV-2 templates. Labour-intensive and time-consuming features of conventional RT-PCR-based sensoring techniques makes them not suitable for rapid and efficient diagnosis. 

Zhao et al. demonstrates a promising alternative to this approach by using poly (amino ester) with carboxyl groups (PC)-coated magnetic NPs (pcMNPs) [125]. The authors successfully fabricated pcMNPs and directly introduced into RT-PCR reaction initiated by combining the lysis and binding step into single step. MNPs were previously reported for their effective application in sensory systems [126]. This method can purify viral RNA from multiple samples within 20 min and 10-copy sensitivity and a strong linear correlation between 10 and 10^5^ copies of SARS-CoV-2 pseudovirus particles. These results are very promising since it reduces operational requirements in current molecular diagnosis of COVID-19, which assist early clinical diagnosis of the affected individuals.

Wang et al. reported a rapid and simultaneous detection system for both SARS-CoV-2 and other respiratory viruses using nanopore target sequencing (NTS) [127]. The authors successfully tested 61 nucleic acid samples from suspected COVID-19 cases and confirmed that NTS is efficient in identifying positive cases within 6 to 0 h. NTS is based upon amplification of 11 virulence-related and specific gene fragments (orf1ab) of SARS-CoV-2 using a primer panel, followed by sequencing the amplified fragments on a nanopore platform. The authors focused on the virulence region (genome bp 21,563–29,674; NC_045512.2), encoding S (1273 amino acids; AA), ORF3a (275 AA), E (75 AA), M (222 AA), ORF6 (61 AA), ORF7a (121 AA), ORF8 (121 AA), N (419 86 AA), and ORF10 (38 AA) proteins. We also considered the RNA-dependent RNA polymerase (RdRP) region in orf1ab. This work is very significant since it provides valuable insights for developing sensory systems for other respiratory pathogens.

Yu et al. also employed the NTS method to detect the alterations of gut microbiota homeostasis in COVID-19-affected individuals [128]. There is huge urgency for developing a potential and rapid nanosensor for detecting SARS-CoV-2 due to the time-consuming process of conventional diagnosis tests. Recently, reports are available claiming the development of rapid detection of recent COVID-19 infections by using COVID-19 IgG-IgM combined antibody colloidal Gold Method Tests [129]. These are a perfect example of “lab to real” transition of useful experimental results and a very optimistic.

## 7. Safety and Limitations

The NPs-based delivery approaches have exhibited significant potential applications, however, studies have showed that these approaches could cause severe damages in respiratory sites and even could impair lung function. The four major patho-biological aspects, including oxidative stress, genotoxicity, inflammation, and fibrosis must be considered with the context of NPs and associated approaches. 

The oxidative stress typically arises due to disparity amongst the reactive oxygen species (ROS) production and the capability of a biological system to voluntarily eradicate the reactive moieties. Sometimes, it could be caused directly due to generation of ROS within the cell or indirectly disturbing the mitochondria respiration [130] or reduce the antioxidant moieties inside the cell environment [131]. The hindrance of oxidative stress might act as an significant phase in initiating few harmful patho-biological activities within cellular microenvironment [132]. Moreover, the effect of NPs over the oxidative stress conditions in the animal models or at cellular level is usually considered as common endpoint studies to detect the toxicity profile of NPs. Both in vivo and in vitro studies play crucial role in understanding the mechanisms of NPs causing oxidative stress. For example, studies have showed significant accumulation of titanium dioxide NPs (TiO_2_-NPs) in the lungs of mice after a 90-day successive intra-tracheal administration of TiO_2_ NPs. The TiO_2_-NPs expressively enhanced the accumulation of ROS level, inflammation, lipid peroxidation level, and also reduced the antioxidants competency in the lungs. The NPs could produce ROS followed by oxidation of antioxidant moieties, and thus could influence the respiratory system and associated patho-biological activities including pulmonary inflammation and genotoxicity [133].

In some cases, it has been observed that the NPs, administered through nasal route, caused chronic or acute inflammation-mediated processes such as inclusion of inflammatory cells and proclamation of cytokines [134,135]. In one of the studies, it was noticed that the direct administration of graphene oxide (GO) solution into lungs of C57BL/6 mice caused extreme pulmonary inflammation with alveolar exudate [135]. The NPs are involved in triggering few pro-inflammatory pathways, including mitogen-activated protein (MAP) kinases [136]. The NPs-treated cells have showed an increased level of AP-1 (activator protein-1) transcription factors and NF-κB (nuclear factor kappa enhancer of triggered B cells), thus affecting the DNA transcription, production of cytokines, and survival of cells [137]. 

Genotoxicity, either primary or secondary, is a major concern associated with NPs-mediated delivery systems. The genotoxic moieties or NPs affect directly by binding with the DNA structures or constituent of the cellular division such as microtubule spindle or centromeres [138]. The carbon nanotubes (CNTs) have directly interacted with DNA assemblies [139]. This indicated that CNTs might cause genotoxicity either in vivo (animal models) or at cellular levels. Studies have revealed that the pulmonary administration of multi-walled CNTs caused genotoxicity by inducing chronic inflammation, which led to insistent oxidative stress [140].

Fibrosis is considered as an indicator of accumulation of inhaled NPs in the pulmonary sites and causes uncommon modes of pulmonary inflammation such as eosinophilia [141]. In one of the studies, the inhaled single-walled CNTs caused multifocal granulomatous pneumonia and fibrosis in treated C57BL/6 male mice model [142].

Currently, various enormous and significant efforts are going on to develop a scientific risk management research structures. The United States National Nanotechnology Initiative/Environmental, Health, and Safety Research Strategy is one of the structures that majorly focusses over the establishment of measuring tools which could efficiently determine the physico-chemical properties of nanotechnology-based delivery systems or nanomedicines [143]. Thus, it is very important to focus on the above mentioned patho-biological processes for delineating the limitations and enhancing the safety concerns of NPs-based approaches for the effective management of respiratory tract infections, diseases or disorders.

## 8. Unsolved Concerns and Perspectives

In spite of the global efforts to apprehend the causes and treatment approaches for COVID-19, many concerns still remain indistinct. Among these concerns, the first concern is that one of the report that has disclosed the presence of COVID-19 in the patient’s stool [27]. Although, COVID-19 could be diffused through the fecal-oral route remains indistinct. Secondly, preceding studies have showed that SARS-CoV and other strains of CoV can endure in environmental surfaces and inorganic objects [17,144] yet, the existence of COVID-19 in the environment surfaces have not been reported. Earlier studies have showed that the CoV can be competently deactivated with the use of surface disinfectants including ethanol (62–71%), hydrogen peroxide (0.5%) and sodium hypochlorite (0.1%) within initial minutes (1–2 min), whereas some other biocidal agents such as benzalkonium chloride (0.05–0.2%) and chlorhexidine digluconate (0.02%) were found to be less effective [17]. However, the present exploration of the efficiency of regularly used disinfectants against COVID-19 is unclear. Thirdly, while the restrictions in travelling were applied in numerous countries, and whether this interference was effective or not, is still unclear. Fourthly, as of now, one case in US has showed a positive response towards remdesivir [27] and also in one of the in vitro study, remdesivir and CQ were found to be an effective therapeutic combination for the management of COVID-19 [33], further clinical trials on the efficiency of remdesivir and CQ for the management of SARS-CoV-2 or COVID-19 should be accomplished for clarity of mechanism. Lastly but most importantly, although numerous studies have been demonstrated with the clinical symptoms of COVID-19 pneumonia in patients of Wuhan and Beijing, which were effectively treated, but as of now there is no specific and effective treatment available worldwide [9,145].

To summarize, these days a dramatic situation on the planet exists. The invisible enemy with the abbreviated name “COVID-19 pandemic” threatens the global health, causing pneumonia induced death in approximately 7% of infected individuals (data up to now, which unfortunately are changing in higher levels). The pathogen causing COVID-19 is SARS-CoV-2, which is believed to be originated from the Wuhan Province in China. Until now, there is no drastic, effective, or successfully applied recipe to face SARS-CoV-2 virus. However, in the last days, there has been a strong effort of R&D of many pharmaceutical industries to discover the appropriate anti-virus cocktail of the already discovered drugs or find a new one. In the whole situation, the impact of nanoscience (nanochemistry, nanomedicine) is significant, but only few scientists worldwide have published their research findings. The latter was due to some preliminary few tests and results exported from animals and in further fewer test in the hospitalized infected patients. It is considered that the first (validated) vaccine can be shared in market during the next year, but until then, all efforts must be done to discover a drastic drug so as to face this invisible enemy, COVID-19.

The most promising statement of the few last days has come from Chinese scientists who claimed to have developed a new weapon to combat COVID-19. They say that they have found a nanomaterial that can absorb and deactivate the virus with 96.5% to 99.9% efficiency [145].

## 9. Conclusions

Global health is facing the most dangerous situation regarding the novel severe acute respiratory syndrome called coronavirus 2 (SARS-CoV-2), which is widely known as the abbreviated COVID-19 pandemic. In this review, the state-of-the-art drug design strategies against the virus are critically summarized including exploitations of novel drugs and potentials of repurposed drugs. The applications of nanochemistry can be summarized in the use of some specific nanomaterials as (i) polymeric, (ii) self-assembling proteins, (iii) inorganic, (iv) peptide-based. Also, special attention is given to NPs-based biosensors, which are vital tools that contribute to the detection of individuals affected with pathogens. Conventional detection methods rely on nucleic acid detection and have various demerits, which are (i) low sensitivity and laborious experimental procedures, (ii) large time duration between sample collection and results interpretation, (iii) high false negative rates, and (iv) lack of specificity which results in misdiagnosis in patients having other viral infections. Some studies have showed that these approaches of nanotechnology could cause severe damages in respiratory sites and even could impair lung function. The four major patho-biological aspects, including oxidative stress, genotoxicity, inflammation, and fibrosis, must be considered with the context of NPs and associated approaches. So, the basic conclusion is that COVID-19 is a new pandemic, and can be initially faced with some already known nanomaterials, which have been applied to the previous SARS-CoV or similar viruses. This knowledge will be a significant tool the fight for this new virus.

## Figures and Tables

**Table 1:** Role of anti-rheumatic drugs in COVID-19 infection [71].

Drug		Mechanism of Action
Chloroquine	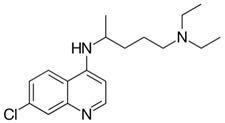	Anti-viral effect via increase of endosomal pH required for virus/cell fusion inhibition of toll-like receptor
Hydroxychloroquine	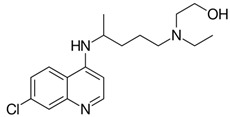	Activity interference with terminal glycosylation of the cellular receptor ACE 2
IL-6 inhibitors		Treatment of cytokine storm manifestations during ARDS
Baricitinib	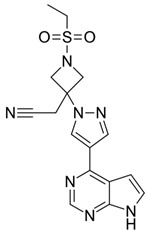	Interference with viral penetration into the cell by blocking NAK-mediated endocytosis Treatment of cytokine storm manifestations during ARDS
TNF-inhibitors		Interference with viral penetration into the cell

**Table 2:** Types of nanoparticles against respiratory viruses [78].

Material	Size (nm)	Virus	Antigen/Epitope	Adjuvant	References
**Polymeric nanoparticles**					
PLGA ^a^	225.4	Bovine parainfluenza 3 virus (BPI3V)	BPI3V proteins	-	[101]
		Swine influenza virus (H1N2)			
	200–300		Inactivated virus H1N2 virus	-	[102]
γ- PGA ^b^	100–200	Influenza (H1N1)	Hemagglutinin	-	[103]
Chitosan	140	Influenza (H1N1)	H1N1 antigen	-	[104]
	300–350	Influenza (H1N1)	HA-Split	-	[105]
	571.7	Swine influenza virus (H1N2)	Killed Swine influenza	-	[106]
	200–250	Influenza (H1N1)	Antigen M2e	Heat shock protein 70 °C	[107]
HPMA/NIPAM ^c^	12–25	Respiratory syncytial virus (RSV)	F protein	TLR-7/8 agonist	[108,109]
Polyanhydride	200–800	Respiratory syncytial virus (RSV)	F and G glycoproteins	-	[110,111]
**Self-assembly proteins and peptide-based nanoparticles**					
N nucleocapside protein of RSV	15	Respiratory syncytial virus (RSV)	RSV phosphoprotein	R192G	[112]
	15	Respiratory syncytial virus (RSV)	Fsll	Montanide ™ Gel01	[113]
	15	Influenza (H1N1)	Antigen M2e	Montanide ™ Gel01	[114]
Ferritin	12.5	Influenza (H1N1)	Antigen M2e	-	[115]
Q11 peptide ^d^	-	Influenza (H1N1)	Acid polymerase	-	[116]
**Inorganic nanoparticles**					
Gold	12	Influenza	Antigen M2e	CpG	[117]
Other					
Virus-like particles (VLP)	80–120	Influenza (H1N1)	Hemagglutinin	-	[118]
	80–120	Influenza (H1N1, H3N2, H5N1)	Antigen M2e	-	[119]
	80–120	Respiratory syncytial virus (RSV)	F protein et G glycoprotein of RSV and M1 protein of influenza	-	[120]
ISCOM ^e^	40	Influenza (H1N1)	Hemagglutinin	ISCOMATRIC	[119]
DLPC liposomes ^f^	30–100	Influenza (H1N1)	M2, HA, NP	MPL and trehalose 6,6′ dimycolate	[121]

^a^ poly(lactic-co-glycolic acid); ^b^ poly-γ-glutamic acid; ^c^ N-(2-hydroxypropyl) methacrylamide/N-isopropylacrylamide, ^d^ fibrilizing peptide(Ac-QQKFQFQFEQQ-Am), ^e^ Quillaia saponin, cholesterol, phospholipid, and associated antigen; ^f^ Dilauroylphosphatidylcholine.
